# Perforated sigmoid colon cancer presenting as an incarcerated inguinal hernia: A case report

**DOI:** 10.1016/j.ijscr.2020.05.067

**Published:** 2020-06-06

**Authors:** Hassan Sabra, Mersad Alimoradi, Etienne El-Helou, Rawan Azaki, Maysaloun Khairallah, Tony Kfoury

**Affiliations:** a Mount Lebanon Hospital, Department of General Surgery, Mount Lebanon, Lebanon; b Lebanese University, Faculty of Medical Sciences, Department of General Surgery, Mount Lebanon, Lebanon

**Keywords:** Colorectal cancer, Perforated colon cancer, Inguinal hernia, Incarcerated hernia

## Abstract

**Background:**

Inguinal hernias are common among the population and usually contain part of the omentum or small bowel, however, other unusual contents have been reported. We report a case of a patient who presented for an incarcerated left inguinal hernia and was found to have an underlying perforated sigmoid cancer.

**Summary:**

An 87 years old man presented with typical signs of an incarcerated left inguinal area. During surgery, we dissected free a herniated hard inflammatory mass until it was found to be covering an underlying perforated sigmoid tumor. Inguinal hernia is a common condition affecting many individuals, and colorectal cancer is one of the rare contents reported inside these hernias. Due to the rarity of such a presentation, the surprise encounter during groin surgery may lead to suboptimal treatment. Perforated colorectal cancer, like in our case, may signify a more aggressive disease, and hence a correct diagnosis is crucial to improve outcomes. When underlying colon cancer is suspected during hernia surgeries, proper exploration and oncologic resection might be the optimal choice.

**Conclusion:**

Surgeons should keep the possibility of underlying colorectal cancer in mind when operating on inguinal hernias and opt for oncologic resection of the identified tumor along with proper lymph node dissection.

## Highlights

• Colorectal cancer can infrequently present as inguinal hernia.
• Perforated colorectal cancer only rarely presents as incarcerated inguinal hernia, like our case.
• When presenting with perforation, colorectal cancer tends to have a worse prognosis.
• When encountered during hernia surgery, surgeons should attempt oncologic resection.

## 1 Introduction

Inguinal hernia is a common condition that occurs when all or part of an internal organ or tissue bulges through the abdominal muscle wall. In most cases, inguinal hernias are manually reducible and asymptomatic, however, a small percentage can present with incarceration [1]. Diagnosis of inguinal hernia is usually done clinically, and imaging is reserved for more complicated cases.

Several uncommon findings have been reported in inguinal hernia sacs, including malignancies, which can be classified into saccular, intrasaccular, or extrasaccular depending on their location [2]. Colorectal cancer (CRC) can occassionaly present as an inguinal hernia [3]. Since such a presentation is rare, and the diagnosis is usually done intraoperatively,there is still no consensus on the best treatment modality for such patients.

We present a case where a contained perforation of an undiagnosed sigmoid colon cancer led to the incarceration of the overlying omentum within a left inguinal hernia, revealing the underlying malignancy intraoperatively.

This case was reported in line with the SCARE criteria [4]

## 2 Case description

An 87-year-old man with a non-significant medical history presented to the emergency department complaining of a painful left inguinal bulge that started one day before presentation. He denied any other complaints. Upon presentation, the patient had a temperature of 38.9 C (102.02 F) but was hemodynamically stable. On physical examination, he had a soft abdomen with an obvious left inguinal bulging that was not manually reducible, very tender to palpation, and had erythematous overlying skin.

**Image 1:**
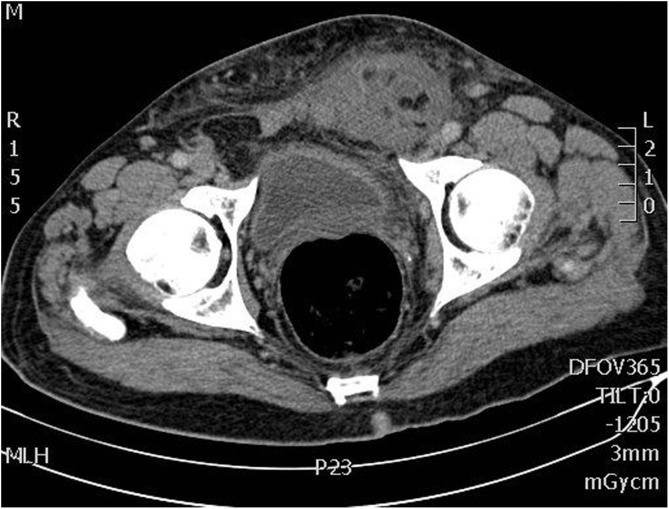
CT Scan revealing a left incarcerated inguinal hernia.

The patient was regarded as having an irreducible left inguinal hernia and routine labs were ordered. A CT scan showed a left fat-containing strangulated inguinal hernia with extensive fat stranding. The sigmoid colon showed focal wall thickening as it passed in proximity to the inguinal hernia (Image 1) Label not found for Float Element. No signs of bowel suffering or perforation were seen. Analgesics and antibiotics were started, and the patient was prepared for open repair of left inguinal hernia.

**Image 2:**
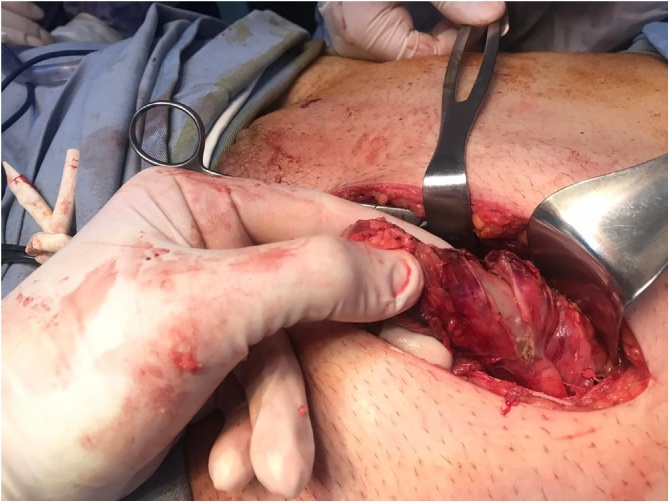
The protruding hard inflammatory mass encountered in the hernial sac.

**Image 3:**
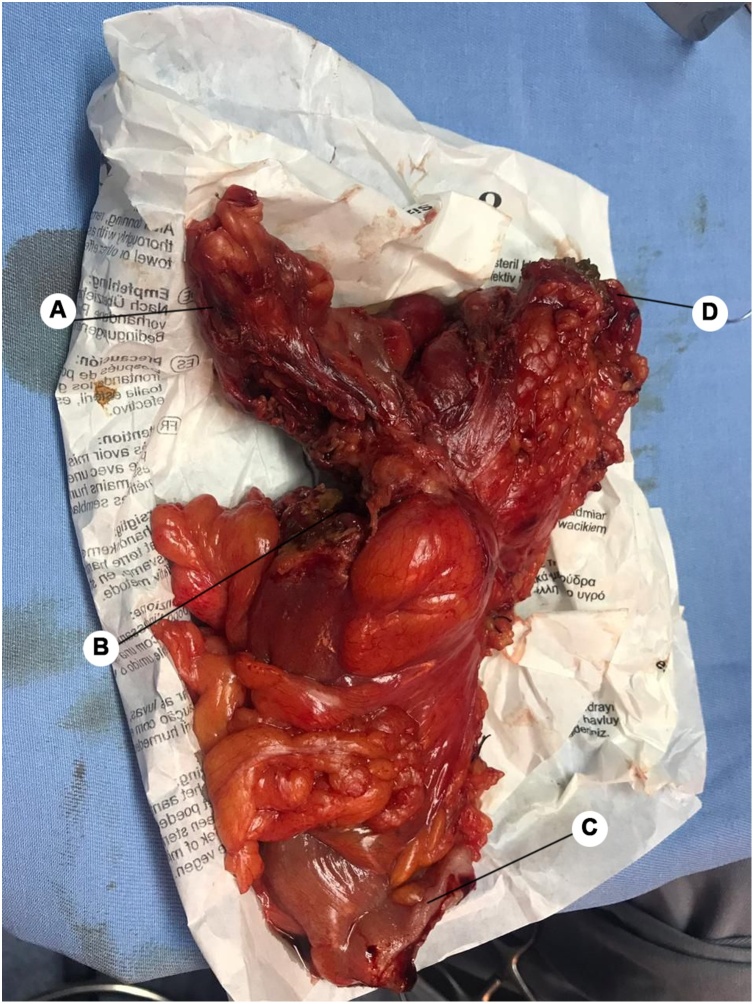
Resected sigmoid. (A) Herniated omental tissue. (B) Perforation site. (C) Inferior resection border. (D) Superior resection border.

During surgery, the abdominal wall aponeurosis was dissected until reaching the hernia. A hard protruding mass was palpated through a tight posterior wall defect with surrounding inflammatory discharge (Image 2) Label not found for Float Element. The peritoneal sac was opened and dissection was done, revealing a hard mass of inflammatory tissue protruding from inside. The incision was extended a few centimeters superiorly, and deeper dissection was performed following the course of the protruding inflammatory mass until it was found to be covering a perforated sigmoid colon. The respective wall of the sigmoid was markedly thickened. These findings were considered suspicious of a neoplastic process, so we decided to treat it as such. Careful exploration and identification of the tumor margins were done (Image 3), followed by resection of the involved sigmoid colon and creation of a colostomy (Hartmanns procedure). Subsequent harvesting of the surrounding lymph nodes was performed. The hernia was then repaired by primary closure without using a mesh due to the contaminated nature of the operation. The patient tolerated the surgery well and was discharged with no complications after a few days.

One week later, pathology reported moderately differentiated infiltrating adenocarcinoma in the resected section of the sigmoid. The surgical margins were negative, however, 7 of the 15 excised lymph nodes were positive for metastasis. The wall of the resected sigmoid showed vascular emboli and signs consistent with perforation.

The patient was referred to the oncology team for treatment planning. Outpatient imaging showed multiple metastatic liver lesions. Chemotherapy was started with the FOLFOX regimen for the first five months, however, the liver metastasis increased on repeat imaging, so he was switched to the FOLFIRI regimen, along with bevacizumab. During this period, and up to the time of writing of this report, the patient did not show any signs of clinical deterioration.

## 3 Discussion

Groin hernia is a very common condition and constitutes the third leading gastrointestinal cause of outpatient visits [5], with a prevalence of 5 %–10 % in the general population [6]. The vast majority of groin hernias are inguinal hernias (96 %), and the remaining are femoral hernias [7]. In most cases, the hernia is reducible, asymptomatic and has a benign course with a relatively low risk of incarceration or strangulation [1]. Diagnosis is usually done clinically without the need for imaging. In complicated cases like ours, imaging with ultrasonography or CT may be indicated. Imaging should be considered when the diagnosis is not obvious to rule out other groin and abdominal pathologies, and to identify hernia complications such as incarceration, strangulation, sepsis, peritonitis, or bowel obstruction. The erythema and sudden onset of pain in our patient raised suspicion of an incarcerated hernia, justifying the need for scanning, however, the radiologist was not able to identify the perforation, and only the incarcerated tissue was seen. Inguinal hernias usually contain part of the omentum or the small bowel, however, some unusual reported findings include an inflamed appendix, diverticulitis, urinary bladder, ovary, colon and, rarely, malignant lesions [8,9].

Neoplastic tumors found in hernias can be classified into three groups: saccular, intrasaccular, and extrasaccular. Saccular tumors are tumors originating from the sac itself such as mesotheliomas. Extrasaccular tumors are tumors present in proximity to the hernia sac, however, they are not contained within it. The last type, intrasaccular tumors, are tumors originating from adjacent tissue and are contained within the sac itself. The latter group most often originates from colorectal cancer [2].

Colorectal cancer most commonly presents with abdominal pain, change in bowel habits, melena, and systemic manifestations of malignancy [10]. However, an infrequent presentation is seen in one in every 200 patients with CRC, where the tumor extends to herniate through an inguinal wall defect [3], with the sigmoid being the part of the colon most commonly implicated with this presentation [9]. Such a diagnosis can be missed by the surgeon, which might lead to incomplete excision of the herniated neoplasm and a delay in diagnosis and treatment.

It is estimated that 3 %–10 % of patients with CRC present with perforation. This presentation is associated with more advanced disease and, therefore, any delay in diagnosis and management of the underlying malignancy might worsen the prognosis [11]. More acutely, the condition can lead to peritonitis and sepsis or even progress into necrotizing fasciitis [12].

In our patient, we believe that an acute contained perforation of the sigmoid tumor developed one day before the presentation, leading to severe inflammation and further herniation of the overlying omentum anteriorly through the inguinal defect with subsequent incarceration due to the evolving inflammation and ischemia. To the best of our knowledge, perforated CRC causing an incarcerated inguinal hernia has only been reported in the English literature twice [12,13], making such a presentation of CRC extremely rare.

In the case of colon cancer perforation, current literature emphasizes on giving priority to sepsis control, especially in unstable patients, where a damage control approach is recommended. Sigmoidectomy should be carried according to the oncological principles, with or without anastomosis and/or stoma formation [14]. The crucial step is to cut out the denominated vessels supplying the tumor-bearing portion at their origin and perform a sufficient lymphadenectomy of more than 12 lymph nodes [15]. In patients in whom less than 12 lymph nodes are resected, adjuvant chemotherapy may be warranted even if no metastatic disease was identified on the resected specimen. Boundaries of resection should be histologically free of tumor cells 5 cm proximally and 1–2 cm distally [16].

## 4 Conclusion

One of the unusual presentations of colorectal cancer is herniation through an inguinal defect. In our case, a contained perforation of an undiagnosed sigmoid cancer lead to inflammation of the overlying mesentery causing an incarcerated inguinal hernia. The presentation is rare, and surgeons should keep the possibility of malignancy in mind while operating on inguinal hernias to avoid suboptimal treatment and delay in diagnosis. When encountered, oncologic resection of the tumor along with at least 12 lymph node excision might be the best choice.

## Declaration of Competing Interest

This article has no conflict of interest with any parties.

## Sources of funding

This research did not receive any specific grant from funding agencies in the public, commercial, or not-for-profit sectors.

## Ethical Approval

The study type is exempt from ethical approval.

## Consent

Written informed consent was obtained from the patient for publication of this case report and accompanying images.

## Author contribution

Writing the paper: Hasan Sabra, Mersad Alimoradi.

Data collection: Etienne El-Helou, Maysaloun Kharallah, Rawan Azaki.

Supervision: Tony Kfoury.

## Registration of research studies

1 Name of the registry: N/A.
2 Unique identifying number or registration ID: N/A.
3 Hyperlink to your specific registration (must be publicly accessible and will be checked): N/A.

## Guarantor

Dr. Tony Kfoury.

## Provenance and peer review

Editorially reviewed, not externally peer-reviewed.
